# Poly(N-vinylpyrrolidone)–Laponite XLG Nanocomposite Hydrogels: Characterization, Properties and Comparison with Divinyl Monomer-Crosslinked Hydrogels

**DOI:** 10.3390/polym14194216

**Published:** 2022-10-08

**Authors:** Ionela Alice Podaru, Paul O. Stănescu, Raluca Ginghină, Ştefania Stoleriu, Bogdan Trică, Raluca Şomoghi, Mircea Teodorescu

**Affiliations:** 1 Department of Bioresources and Polymer Science, Faculty of Chemical Engineering and Biotechnologies, Politehnica University of Bucharest, 1–7 Gh. Polizu Str., 011061 Bucharest, Romania; 2 Armament Systems and Mechatronics Department, Military Technical Academy “Ferdinand I”, 39–49 G. Cosbuc Blvd., 050141 Bucharest, Romania; 3 Advanced Polymer Materials Group, Politehnica University of Bucharest, 1–7 Gh. Polizu Str., 011061 Bucharest, Romania; 4 Chemical Technologies for CBRN Defense Department, Research and Innovation Center for CBRN Defense and Ecology, 225 Olteniţei Ave., 041327 Bucharest, Romania; 5 Department of Science and Engineering of Oxide Materials and Nanomaterials, Faculty of Chemical Engineering and Biotechnologies, Politehnica University of Bucharest, 1–7 Gh. Polizu Str., 011061 Bucharest, Romania; 6 National Institute for Research and Development in Chemistry and Petrochemistry—ICECHIM, Spl. Independentei 202, 060021 Bucharest, Romania; 7 Faculty of Petroleum Technology and Petrochemistry, Petroleum and Gas University of Ploiesti, 39 Bucuresti Blvd., 100680 Ploiesti, Romania

**Keywords:** poly(N-vinylpyrrolidone), nanocomposite hydrogel, Laponite, N,N′-methylene-bisacrylamide, tri(ethylene glycol) divinyl ether

## Abstract

The present work investigates, for the first time, the synthesis and properties of some nanocomposite (NC) hydrogels obtained by the aqueous solution free radical polymerization of N-vinylpyrrolidone (NVP) in the presence of Laponite XLG (XLG) as a crosslinker, in comparison with the corresponding hydrogels prepared by using two conventional crosslinking divinyl monomers: N,N′-methylenebisacrylamide (MBA) and tri(ethylene glycol) divinyl ether (DVE). The structure and properties of the hydrogels were studied by FTIR, TEM, XRD, SEM, swelling and rheological and compressive mechanical measurements. The results showed that DVE and XLG are much better crosslinking agents for the synthesis of PNVP hydrogels than MBA, leading to larger gel fractions and more homogeneous network hydrogels. The hydrogels crosslinked by either DVE or XLG displayed comparable viscoelastic and compressive mechanical properties under the experimental conditions employed. The properties of the XLG-crosslinked hydrogels steadily improved as the clay content increased. The addition of XLG as a second crosslinker together with a divinyl monomer strongly enhanced the material properties in comparison with the hydrogels crosslinked by only one of the crosslinkers involved. The FTIR analyses suggested that the crosslinking of the NC hydrogels was the result of two different interactions occurring between the clay platelets and the PNVP chains. Laponite XLG displayed a uniform distribution within the NC hydrogels, the clay being mostly exfoliated. However, a small number of platelet agglomerations were still present. The PNVP hydrogels described here may find applications for water purification and in the biomedical field as drug delivery systems or wound dressings.

## 1. Introduction

Hydrogels are chemically or physically crosslinked networks of both synthetic and natural hydrophilic polymers; they are able to absorb and retain large amounts of water and aqueous solutions [1,2,3]. Due to their water content and elasticity, which make them resemble human tissue, hydrogels have many uses in the biomedical field, such as tissue engineering, controlled drug delivery, biosensors, wound management, bioprinting, bioadhesives, etc. [2,4,5,6,7,8,9,10,11,12]. Hydrogels also display applications in numerous other fields, such as agriculture, catalysis, microfluidics, actuators, cosmetics and hygiene products, membranes, water treatment and so on [2,13,14,15,16,17,18,19]. The wide range of applications of the hydrogels owes to the employment of a large number of (co)polymers in the formation of the hydrogel network. On the other hand, hybrid or (nano)composite hydrogels can be created by combining the hydrogel with additional polymers or (nano)particles. The addition of these components may enhance the current qualities of the hydrogel and/or impart new advantageous traits, broadening its potential applications.

The nanocomposite hydrogels field is a rapidly expanding one because some of the hydrogel properties, such as the mechanical, optical, electrical, adsorption or thermal ones, may be improved, while some new ones may be brought to the material by adding various carbonaceous (carbon nanotubes, graphene oxide), metallic (Au, Ag), metal oxide (alumina, Fe_2_O_3_) or layered clays (Laponite, Montmorillonite) nanoparticles, among others [9,18,19,20,21]. Among the nanocomposite hydrogels, a special place is dedicated to those that employ Laponite as the crosslinker and which are usually denoted as “NC” gels/hydrogels [22,23]. 

The NC hydrogels were first reported by Haraguchi and Takehisa [24], who free-radically polymerized N-isopropylacrylamide in the presence of Laponite XLG as the only crosslinking agent, resulting in stable hydrogels with remarkable mechanical and optical properties, in contrast to their N,N′-methylenebisacrylamide-crosslinked counterpart [22,24,25]. Since then, numerous NC hydrogels have been prepared, mostly from acrylamide-type monomers, such as N-isopropylacrylamide [24,26,27,28], acrylamide [29,30,31], N,N-dimethylacrylamide [29,31,32,33], N,N-diethylacrylamide [34], N-acryloyl glycinamide [35], 4-acryloylmorpholine [36] and their copolymers with monomers such as 2-methoxy-ethyl acrylate [37], sodium methacrylate [38,39,40] and acrylate [41], 2-(dimethylamino)ethyl methacrylate [42] and isocyanoethyl methacrylate-glutamine [43]. NC hydrogels from non-acrylamide monomers have been reported in only very few cases: N-vinylpyrrolidone [44,45], 2-dimethylaminoethyl methacrylate [46] and 2-(2-methoxyethoxy)ethyl methacrylate–oligo(ethylene glycol) methacrylate copolymer [47]. Both Laponite XLG/RD [24,31,33,38,40] and Laponite XLS/RDS [27,29,30,31,42] were used as crosslinking agents, while the polymerization was initiated most often by the potassium peroxodisulfate-N,N,N′,N′-tetramethylethylenediamine redox initiating system [24,26,28,29,30,31,32,33] and in a much lower extent by 2,2′-azobis(isobutyronitrile) [33,44] and photoinitiators [27,35,36].

Laponite is a synthetic clay structurally similar to natural hectorite, having the empirical formula Na^+^_0.7_[(Si_8_Mg_5.5_Li_0.3_)O_20_ (OH)_4_]^−0.7^ [48]. Laponite dry powder contains crystalline stacks which convert to isolated discs that are about 25 nm in diameter and 0.92 nm thick when dispersed in water. The discs contain weak positive charges on the edges and negative charges on the surface, leading to an electrostatic self-assembly of the discs in water, resulting in a house-of-cards-type structure in the case of unmodified Laponite XLG/RD. The formation of this structure produces gelation if the aqueous dispersion concentration is larger than 2% [48]. By neutralizing the Laponite positive charges with pyrophosphate anions (Laponite XLS/RS), the formation of the house-of-cards structure is avoided, and larger amounts of clay can be dispersed in water without gel formation [49].

The NC hydrogels structure, which was studied in the context of acrylamide-type monomers, is made up of a network of polymer chains that act as bridges and individual Laponite discs that serve as crosslinking points. Each clay disc is wrapped in a layer of several polymer chains, each of which has multiple interaction sites with the disc surface. The layer is about 1 nm thick. The polymer chain–clay interaction was assumed to occur through hydrogen bonds between the amide group of the monomer units within the polymer and the Si-O moieties on the clay platelet surface. Most polymer chains connect at least two discs, but there are also dangling chains [25,26].

The network formation starts in the vicinity of the Laponite platelets, where initiator and monomer molecules are adsorbed. The macroradicals grow from the platelet until a chain-breaking reaction (termination, chain transfer) with another species existing in the polymerization medium occurs. If termination by combination between two macroradicals grafted on different discs takes place, a polymer bridge between those two discs (effective network chain) forms. In all the other cases, the result is just polymer chains connected at only one end (dangling chains) to the clay disc or loops. Large finite microgel clusters form at the beginning, which further connect each other, resulting in hydrogel formation [25,26].

Poly(N-vinylpyrrolidone) (PNVP), which can be synthesized by the free-radical polymerization of N-vinylpyrrolidone (NVP), is a biocompatible, neutral and non-toxic hydrophilic polymer that is soluble in water and organic solvents, is able to complex both hydrophilic and hydrophobic compounds and has numerous applications [50,51,52]. Its hydrogel derivatives have been obtained either by NVP polymerization in the presence or absence of a divinyl comonomer or by crosslinking the preformed PNVP through irradiating its aqueous solutions with high energy or UV rays, usually in the presence of another polymer to enhance the mechanical properties of the hydrogel product [13,50,51,53,54]. Due to the remarkable properties of the constituent polymer, PNVP hydrogels display many (potential) applications such as wound management [4], disintegrants and dissolution agents in pharmaceutical tablets [55], drug [56] and protein [57] delivery systems, water purification [44,58], etc.

Surface and ground water pollution is an important issue of the present days, and from this point of view, (nano)composite hydrogels represent a valuable treatment solution for the contaminated waters [18,19]. This should be especially true when both the polymer and the filler display complexing/adsorption ability for various chemicals acting as water pollutants, such as in the case of poly(N-vinylpyrrolidone) NC hydrogels [44]. The present work aims at investigating, for the first time, the synthesis and properties of the hydrogels obtained by the aqueous solution free radical polymerization of NVP in the presence of Laponite XLG as a crosslinker, in comparison with the corresponding hydrogels prepared by using two conventional crosslinking divinyl monomers: tri(ethylene glycol) divinyl ether (DVE) and N,N′-methylenebisacrylamide (MBA). NVP is not an acrylamide-type monomer because the vinyl group is linked to the nitrogen atom of the amide moiety instead of the carbonyl group (Figure S1, Supplementary Data file), but it is still able to form NC hydrogels. To the best of our knowledge, there are only two papers in the literature reporting the preparation of a poly(N-vinylpyrrolidone) NC hydrogel [44,45], but no such a comparison has been shown by now. The present paper also reports, for the first time, the use of DVE as the crosslinking monomer for the free radical polymerization synthesis of PNVP hydrogels, both regular and NC, and its benefits as far as the PNVP hydrogel synthesis and properties are concerned, in comparison MBA as a crosslinker. We will show within this paper that DVE and Laponite XLG are much better crosslinking agents for the synthesis of PNVP hydrogels than MBA, leading to larger gel fractions and more homogeneous network hydrogels. Additionally, the hydrogels crosslinked by either DVE or XLG displayed comparable viscoelastic and compressive mechanical properties under the experimental conditions employed, while the addition of XLG as a second crosslinker together with the divinyl monomer strongly enhanced the material properties in comparison with the hydrogels crosslinked by only one of the crosslinkers involved. We will also point out by FTIR analysis that the crosslinking of the NC hydrogels is the result of two different interactions occurring between the clay platelets and the PNVP chains.

Besides water purification, the PNVP hydrogels described here may also find other applications, such as in the biomedical field as drug delivery systems or wound dressings.

## 2. Materials and Methods

### 2.1. Materials

N-Vinyl-2-pyrrolidone (NVP, ACROS Organics, Geel, Belgium, 98%) and tri(ethylene glycol) divinyl ether (DVE, Aldrich, St. Louis, MI, USA, 98%) were distilled under vacuum and stored in the freezer. Azobisisobutyronitrile (AIBN, Fluka, Buchs, Switzerland, 98%) was recrystallized from methanol and stored at 4 °C. N,N′-Methylenebisacrylamide (MBA, Merck, Hohenbrunn, Germany, 98%) and Laponite XLG (XLG, BYK Additives & Instruments, kindly provided by Cosichem & Analytical, Bucharest, Romania) were used as received. Distilled water (DW) was employed in all experiments involving water.

### 2.2. Hydrogel Preparation

The hydrogels were prepared either in glass molds in order to obtain hydrogel discs to be used for rheological measurements or in 9 mm inner diameter cylindrical glass tubes in order to obtain cylindrical hydrogel samples for compressive mechanical tests. To obtain the hydrogels, the appropriate amounts of DW and XLG (Table 1) were magnetically stirred at room temperature for 2 h to exfoliate the clay in the first step. Next, MBA (4 mol% based on NVP) was added to the clear dispersion in the case of MBA-crosslinked hydrogels, and after the full dissolution of MBA, an AIBN solution (0.25 mol% to NVP) in NVP (20 wt% to the whole polymerization mass) was added. For DVE-crosslinked hydrogels, because of the lower water solubility, DVE was added to the polymerization mixture as a solution in NVP, together with AIBN. Depending on the hydrogel prepared, either the divinyl monomer or XLG may have been omitted (Table 1). After 10 min of stirring, the homogeneous solution was degassed under the vacuum obtained from a water vacuum pump for a few minutes and then transferred via needle and syringe into either a nitrogen-purged glass mold or rubber septum-sealed glass tubes. The mold was made up of two glass plates separated by a 1.4 mm-thick rubber gasket. The glass mold and the glass tubes were then placed in an oven or in an oil bath, respectively, at 50 °C for 22 h.

At the end of the reaction time, the mold was cooled down and disassembled, and discs that were 8 mm and 20 mm in diameter were cut from the hydrogel. The discs were purified by immersing them into excess DW at room temperature for 7 days, with a daily change of water, and then kept in DW at 25 ± 0.5 °C for 3 more days. The swollen discs that were 20 mm in initial diameter were used to obtain 20 mm precise diameter discs, which were immediately employed for rheological measurements, while the purified and swollen 8 mm hydrogel discs were employed to determine the equilibrium swelling degree. To determine the monomer conversion (C) and gel fraction (GF), a certain amount of the remaining as-prepared hydrogel (W_h,0_) was first dried in the atmosphere, when most of the unreacted NVP and water evaporated, and then dried in a desiccator over anhydrous CaCl_2_ to a constant weight (W_x_). The monomer conversion was calculated according to Equation (1).

C (%) = (W_x_ − W_h,0_ × w_xlg_)/(W_h,0_ × w_monomer_) × 100 (1)

where w_xlg_ and w_monomer_ are the mass fractions of the XLG and monomer, respectively, in the initial hydrogel composition.

Next, a weighed amount of NVP-free dry xerogel (W_x,0_) was purified for 7 days in DW with a daily change of water and then dried, first in an oven at 50 °C for a week and then in a desiccator over anhydrous CaCl_2_ to constant weight (W_x,f_). The gel fraction (GF) was calculated by using Equation (2).

GF (%) = W_x,f_/W_x,0_ × 100 (2)

In the case of the hydrogel samples for the compression mechanical tests, the glass tubes kept in the oil bath at 50 °C for 22 h of polymerization time were cooled down and carefully broken, and the resulting hydrogel rods were cut into cylinders that were about 10 mm in height. Small disc-shaped hydrogel pieces that were 1.5–2 mm thick were also collected for NVP conversion, gel fraction and equilibrium swelling determinations, and they were processed as described above. The GF values reported represent the average ± the error between the gel fractions of two equivalent hydrogel samples, one of them prepared by the glass mold procedure and the other one synthesized in a glass tube.

The hydrogel cylinders were purified by immersion in DW at room temperature for 7 days with a daily change of water, kept in DW at 25 °C for 3 more days and then mechanically tested.

### 2.3. Equilibrium Swelling Degree Determination

The purified and swelled hydrogel discs, kept in DW at 25 °C for 3 additional days, were removed from water, superficially wiped with moisturized filter paper and accurately weighed (W_H_). The hydrogels were then dried in an oven at 50 °C for a week, and after that, they were dried in a desiccator over anhydrous CaCl_2_ to constant weight (W_X_). The equilibrium swelling degree (ESD) was calculated according to Equation (3) and reported as the average ± the error between the ESD values obtained for two equivalent hydrogel samples, one of them prepared by the glass mold procedure and the other one synthesized in a glass tube.

ESD = (W_H_ − W_X_)/W_X_ (g water/g xerogel) (3)

### 2.4. Characterizations

Attenuated Total Reflectance–Fourier Transform Infrared (FTIR) analyses were recorded on a Perkin Elmer Spectrum Two (Perkin Elmer, Waltham, MA, USA) instrument with a Pike MiracleTM ATR modulus, with a resolution of 1 cm^−1^ and an accumulation of 32 scans.

Thermogravimetric (TGA) investigations were performed on NETZSCH TG 209F1 Libra equipment by heating xerogel samples of about 2 mg from room temperature to 700 °C at a rate of 10 °C/min under nitrogen flow.

X-ray diffraction (XRD) analyses were carried out on a Shimadzu 6000 diffractometer by using CuK radiation under a voltage of 40 kV and a current of 30 mA. A 0.02° step size and a scanning speed of 2°/min were employed to obtain the XRD patterns. The XRD analyses, as well as the FTIR and TGA ones, were performed on xerogel powders.

Scanning electron microscopy (SEM) micrographs of the previously swollen and freeze-dried (2.5 FreeZone Labconco freeze dryer) hydrogel samples were obtained by using a Tescan Vega II LMU microscope. The EDX microanalysis was performed by using a Bruker Quantax XFlash 6/10 energy dispersive X-ray spectrometer. Elemental mapping and spectrum quantification with P/B-ZAF were performed by means of the Esprit software. Before analysis, the samples were coated with a thin layer of Au/Pd (80/20) alloy under Argon plasma.

Transmission electron microscopy (TEM) images were collected by means of a G2 F20 TWIN Tecnai FEI Company (Eindhoven, The Netherlands) instrument. The TEM samples were prepared by including the xerogel powder in epoxy resin (Agar 100 Resin Kit, Agar Scientific, Stansted, UK), curing the mixture at 60 °C for 48 h and slicing the hard samples to 70 mm-thick pieces at a 2 mm/s cutting speed.

The rheological measurements were performed at 25 °C on a Kinexus Pro rheometer (Malvern Instruments, Malvern, UK, software 1.60) with a Peltier temperature control unit by employing 20 mm parallel plates with rough faces. Hydrogel samples that were 1.5–2.5 mm thick and swelled at equilibrium at 25 °C were analyzed. A 0.3 N normal force was applied in all cases in order to avoid slippage, except for the HXLG_5_ hydrogel, which was too soft to withstand this pressure, and a gap of 2.3 mm was set instead. Amplitude sweep measurements were performed at 1 Hz. A strain within the linear viscoelasticity region was selected to be used in the frequency sweep experiments, which were carried out in the 0.1–10 Hz range by employing the “controlled strain” mode. To prevent water evaporation from the hydrogel during the analysis, a few water drops were placed on the lower plate, and both plates and hydrogel were placed under an insulating cover. Two measurements were carried for each hydrogel sample (two hydrogel discs), and the average values ± the error were reported.

The uniaxial mechanical compressive tests were carried out on equilibrium-swollen cylindrical hydrogel samples by using an Instron 3382 instrument equipped with a 2 kN cell at room temperature, at a 2 mm/min compression rate, until failure. Both the elastic (E) and shear (G) moduli, the ultimate compressive stress (τ_max_), i.e., the highest stress value on the stress–strain curve, and the corresponding ultimate compressive strain (1−λ)_max_, as well as the compressive toughness (T_c_), were calculated from the stress–strain curves and reported. Five measurements were carried out for each sample, and the average values ± the standard deviation were calculated and reported. The elastic (Young’s) modulus was determined from the stress (τ)–strain (1−λ) plots according to Equation (4):

τ = E (1−λ)(4)

where τ represents the force applied to the hydrogel surface unit, while λ is the ratio between the instantaneous (l) and initial height (l_0_) of the cylindrical hydrogel sample. The shear modulus was determined as the slope in τ vs. (λ^−2^−λ) plots, according to Equation (5) [60]:

τ = G (λ^−2^−λ) (5)

Both E and G were determined for strains within the 3–8% range, where the plot was considered linear. The compressive toughness of the hydrogels, i.e., the work to fracture, was calculated as the area under the stress–strain curve before sample fragmentation [61,62].

## 3. Results and Discussion

### 3.1. Synthesis and Structure of Hydrogels

The PNVP hydrogels were obtained by the free-radical polymerization of NVP in the presence of XLG and/or MBA/DVE as inorganic and organic crosslinking agents, respectively, in aqueous solutions and in the presence of AIBN as the initiator. The NVP concentration was fixed at 20 wt% of the whole polymerization mixture, while the amounts of the divinyl monomer and AIBN were set to 4 mol% and 0.25 mol%, respectively, based on NVP (Table 1). When used together with MBA or DVE, XLG was employed in a concentration of 10 wt% to NVP, while 5 wt%, 10 wt% and 15 wt% of XLG with respect to NVP were added when XLG was the sole crosslinking agent (Table 1). Hydrogels were designated by an “H” followed by the abbreviation/abbreviations of the crosslinking agent/agents and some numbers as subscripts indicating the weight (XLG) or mole (MBA/DVE) proportion of the crosslinking agent in relation to NVP. For example, HMBA_4_ and HXLG_10_ denote the hydrogels with MBA (4 mol% to NVP) and XLG (10 wt% to NVP), respectively, as crosslinkers, while HMBA_4_-XLG_10_ stands for the hydrogel crosslinked with both MBA (4 mol% to NVP) and XLG (10 wt% to NVP).

After stirring the clay in DW for 2 h, a clear and colorless dispersion resulted, which was liquid for the compositions with 5 wt% and 10 wt% XLG to NVP, while it displayed the properties of a soft gel in the case of 15 wt% XLG. However, the addition of the NVP solution dissolved the gel, similar to the reported N-isopropylacrylamide effect [63], resulting in a low-viscosity liquid mixture comparable to those obtained for the other hydrogels under the experimental conditions employed. This gel dissolution was previously ascribed to the mild interaction among the monomer molecules and Laponite platelets [39,63]. By increasing the XLG concentration to 30 wt% to NVP and preserving the indicated proportions of the hydrogel precursor solutions, a clay dispersion too viscous to be manipulated was obtained, regardless of whether NVP was added to the dispersion of XLG in DW or, conversely, XLG was added to a solution of NVP in DW.

**Figure 1:**
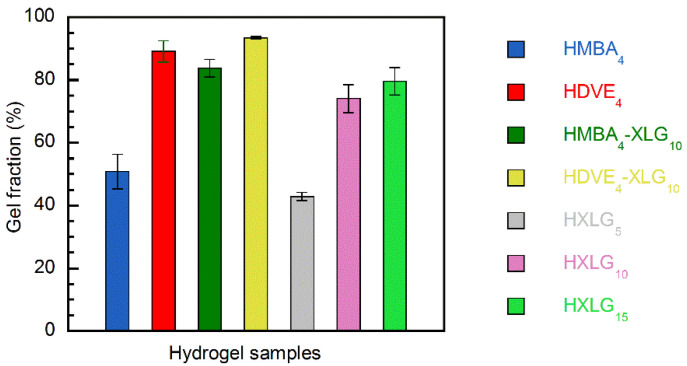
Gel fractions of the hydrogel samples synthesized.

The monomer–crosslinking agent–initiator mixtures were polymerized at 50 °C for 22 h, resulting in very high monomer conversions (91–100%) in all cases. However, the amount of polymer included in the hydrogel network was considerably less than complete, being strongly dependent on the type and amount of the crosslinking agent, as indicated by the gel fractions determined (Figure 1).

When a divinyl monomer was employed as the only crosslinking agent, the gel fraction obtained in the case of MBA (GF = 50.7 ± 5.5%) was much lower than that obtained for DVE (89.0 ± 3.4%). This can be explained by the different reactivities of MBA, DVE and NVP in radical copolymerization. NVP and DVE are non-conjugated vinyl monomers with a lower reactivity in radical copolymerization, while MBA displays a higher reactivity because of its vinyl double bonds being conjugated with the carbonyl groups from the amide moieties (Figure S1). The much higher reactivity of MBA in its copolymerization reaction with NVP, as supported by the copolymerization reactivity ratios of the acrylamide (AM)–NVP system (r_AM_/r_NVP_ = 0.66/0.17 [64], r_AM_/r_NVP_ = 0.61/0.05 [65]), led to a much faster consumption of MBA in comparison with NVP. As a consequence, the gel fraction was only about 50%, although the conversion of monomers to polymer was practically complete, in agreement with previously reported results [66].

**Figure 2:**
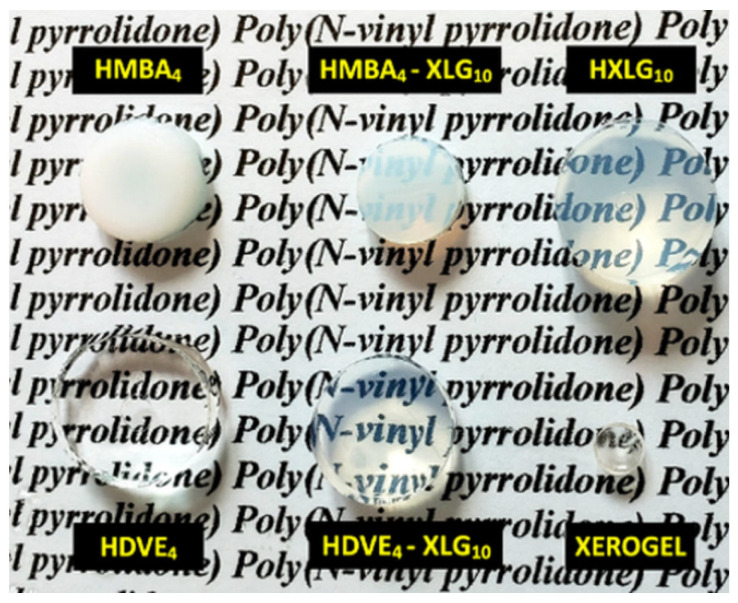
Appearance of the equilibrium-swelled hydrogels.

**Figure 3:**
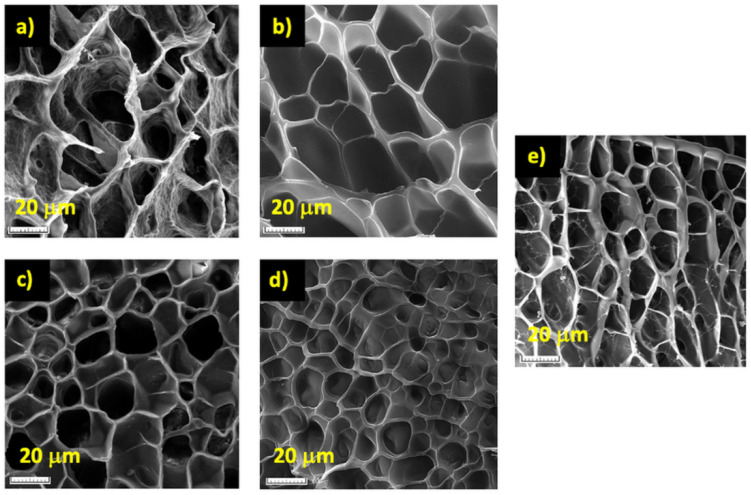
SEM images of lyophilized hydrogels. (**a**) HMBA_4_; (**b**) HDVE_4_; (**c**) HMBA_4_-XLG_10_; (**d**) HDVE_4_-XLG_10_; (**e**) HXLG_10_.

The much higher reactivity of MBA in comparison with NVP also led to the formation of an inhomogeneous gel, made up of more dense regions formed at the beginning of the polymerization process and containing larger amounts of MBA, surrounded by less crosslinked regions of a lower density, which resulted later during the gel formation [30]. The inhomogeneous/multiphasic character of the HMBA_4_ hydrogel was supported by its opaque appearance when swelled in water (Figure 2), as well as by the rough/inhomogeneous appearance of the cell walls of the lyophilized hydrogel, as evidenced by the SEM analysis (Figure 3a).

Unlike MBA, the copolymerization of DVE with NVP led to a much higher gel fraction (Figure 1) due to the much closer reactivities of the two types of vinyl groups, i.e., vinyl ether and vinyl amine (Figure S1), which allowed for the presence of DVE molecules available for crosslinking up to very high monomer conversion. This explanation is supported by the results of Khutoryanskiy and coworkers [67], who showed that, in the case of NVP–vinyl propyl ether (VPE) copolymerization in ethanol for an NVP–VPE feed mixture of 90–10 mol%, a copolymer with an 86.6–13.4 mol% composition at a copolymer yield of about 90% was obtained. Besides the higher gel fraction, the close consumption rates of the two co-monomers, together with the inability of the vinyl ether groups of DVE to free-radically homopolymerize [68], led to a much more homogeneous hydrogel network than that in the case of HMBA_4_, as proven by the full transparency of the equilibrium-swelled HDVE_4_ hydrogel (Figure 2) and the smooth cell walls of the lyophilized material (Figure 3b).

The NVP polymerization in the presence of XLG, without any divinyl monomer added, led to crosslinked materials as well, thus proving that NVP is able to form NC hydrogels, although it is not an acrylamide-type monomer. The gel fraction increased with the XLG concentration (Figure 1), reaching about 80% in the case of HXLG_15_ under the experimental conditions employed. The gel fraction of HXLG hydrogels was lower than that of HMBA_4_ in the case of HXLG_5_, but it overpassed this one by 25–29% when the XLG proportion increased to 10–15 wt% to NVP. As compared with HDVE_4_, the HXLG hydrogels displayed a gel fraction that was lower by at least 10% (Figure 1).

The resulting HXLG hydrogels were slightly cloudy (Figure 2) because of the presence of small amounts of non-exfoliated clay particles, as will be shown below, while the lyophilized material displayed smooth cell walls (Figure 3e). This proved the formation of a homogeneous hydrogel with a uniform distribution of the XLG crosslinking points, similar to the DVE-crosslinked hydrogel.

By combining both XLG and a divinyl monomer as crosslinking agents (HMBA_4_-XLG_10_; HDVE_4_-XLG_10_), the gel fraction increased in comparison with both corresponding hydrogels containing only one crosslinker, i.e., GF_HMBA4-XLG10_ > GF_HXLG10_ > GF_HMBA4_ and GF_HDVE4-XLG10_ > GF_HDVE4_ > GF_HXLG10_ (Figure 1), as expected. The most beneficial effect from this point of view was recorded in the case of MBA as a crosslinker, where its combination with XLG led to a 33% increase in the gel fraction. Owing to the addition of XLG, the equilibrium-swelled HMBA_4_-XLG_10_ hydrogel became slightly transparent (Figure 2), probably because of the densely MBA-crosslinked regions getting smaller and better dispersed within the hydrogel. This supposition was supported by the SEM image of the lyophilized HMBA_4_-XLG_10_ hydrogel (Figure 3c), which showed cell walls that were much less rough than those in the case of HMBA_4_ but also less smooth than those for HXLG_10_ (Figure 3e). Unlike the HMBA_4_-XLG_10_ case, HDVE_4_-XLG_10_ became slightly cloudy through XLG addition because of the non-exfoliated clay particles (Figure 2), while the smooth aspect of the lyophilized sample cell walls was not affected (Figure 3d), indicating that the homogeneous character of the hydrogel network was mostly preserved after the addition of XLG.

**Figure 4:**
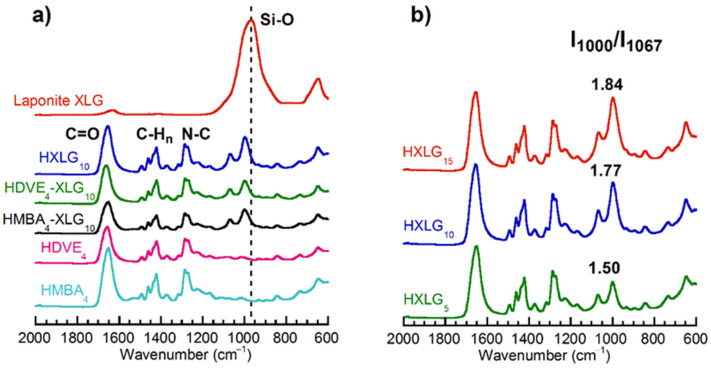
FTIR spectra of the hydrogels synthesized. (**a**) Hydrogels crosslinked with XLG and/or MBA/DVE; (**b**) NC hydrogels crosslinked with various amounts of XLG. I_1000_/I_1067_ is the ratio of the intensities (height) of the bands at 1000 cm^−1^ and 1067 cm^−1^.

The FTIR analysis of the powdered xerogels demonstrated the presence of XLG in the NC hydrogels, as well as the presence of PNVP–XLG interactions occurring within the NC hydrogel matrix (Figure 4). All FTIR spectra displayed the characteristic bands of the PNVP matrix: >C=O (amide I) at ≈1650–1660 cm^−1^, C-H_n_ deformations at ≈1420–1490 cm^−1^ and N-C stretching at ≈1290 cm^−1^ (Figure 4a) [69,70]. Additionally, the Si-O band of XLG at 967 cm^−1^ [71] was found in all NC hydrogel spectra but modified because of the interaction with PNVP (Figure 4a,b). The Si-O band of the clay contained in all NC hydrogels was split into two bands, both shifted to higher wavenumbers: a higher intensity band at 1000 cm^−1^ and a band with a lower intensity at 1066–1070 cm^−1^. A 7 cm^−1^ shift of the carbonyl band was noticed after the addition of XLG together with MBA or DVE in the hydrogel composition (1651 cm^−1^ HMBA_4_ → 1658 cm^−1^ HMBA_4_-XLG_10_; 1655 cm^−1^ HDVE_4_ → 1662 cm^−1^ HDVE_4_-XLG_10_) as well. The shift of both carbonyl and Si-O bands in the NC hydrogels supported the previous supposition that the polymer–clay interaction occurs through hydrogen bonds between polymer amide groups and functional moieties (Si-OH or Si-O-Si) on the XLG surface [25]. The band splitting phenomenon seems to indicate that two different PNVP–clay interactions occurred in the NC hydrogels. According to the intensity of these bands, the proportion of the interactions was different, and it seemed to modify as the XLG concentration in the hydrogel changed (Figure 4b). The FTIR spectra in Figure 4b also demonstrated the increasing clay concentration within the HXLG_5-10-15_ hydrogels, as expected.

**Figure 5:**
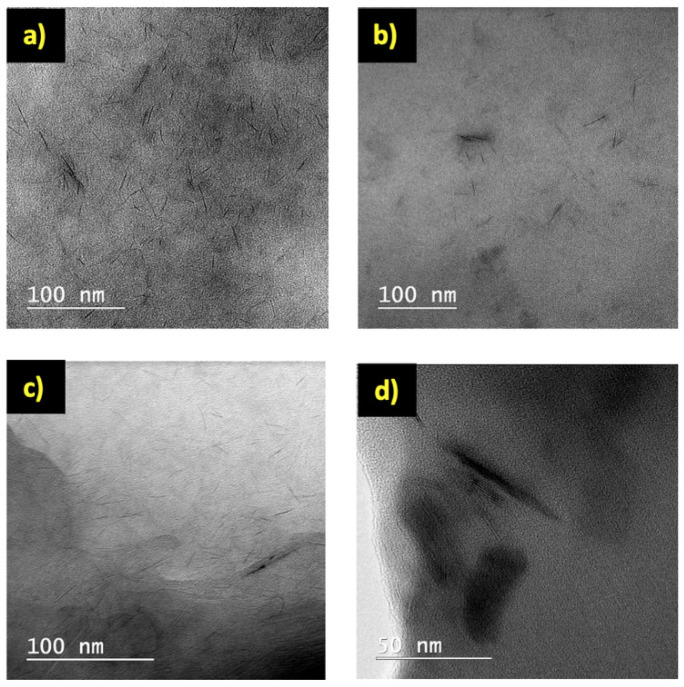
TEM images for the NC hydrogels. (**a**) HMBA_4_-XLG_10_; (**b**) HDVE_4_-XLG_10_; (**c**) HXLG_10_; (**d**) non-exfoliated clay structures in HDVE_4_-XLG_10_.

Besides the FTIR analysis, the presence of XLG within the NC hydrogels was additionally demonstrated by TEM, EDX microanalysis and TGA. The TEM images showed that XLG was mostly exfoliated as single layers with random orientation within the NC hydrogels (Figure 5a–c), but some clay platelet agglomerations (Figure 5a–c), including more organized/non-exfoliated structures (Figure 5d), were still visible.

**Figure 6:**
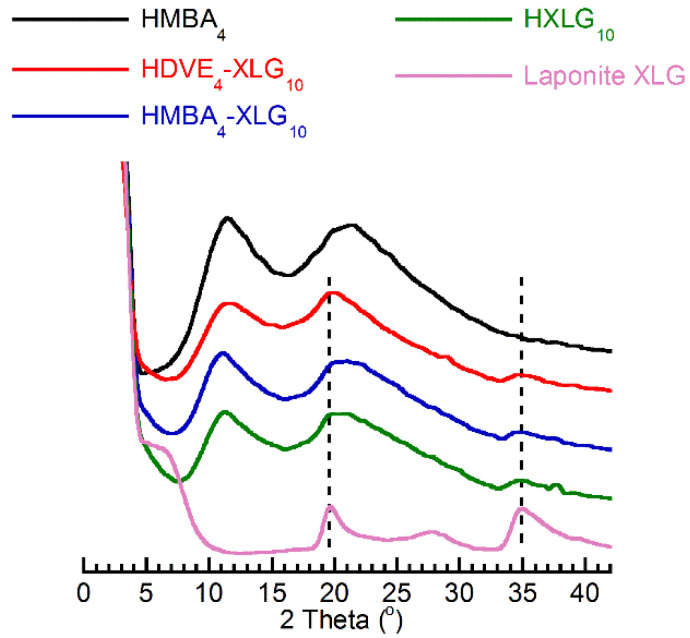
XRD diffractograms of NC hydrogels in comparison with Laponite XLG and the PNVP matrix.

The presence of non-exfoliated clay structures within the NC xerogels was confirmed by the XRD analysis, which evidenced the presence of some small-intensity XLG-belonging peaks on the diffractograms of the NC hydrogels (Figure 6). All hydrogel diffractograms displayed the XRD patterns of the PNVP matrix, i.e., two diffuse peaks at 2θ ≈ 11.5° and 2θ ≈ 21.5°. The first one was previously ascribed to the intermolecular interactions among the C-C polymer backbones, while the second one was ascribed to the inter- and intramolecular interactions between the pendant pyrrolidone rings [72].

**Figure 7:**
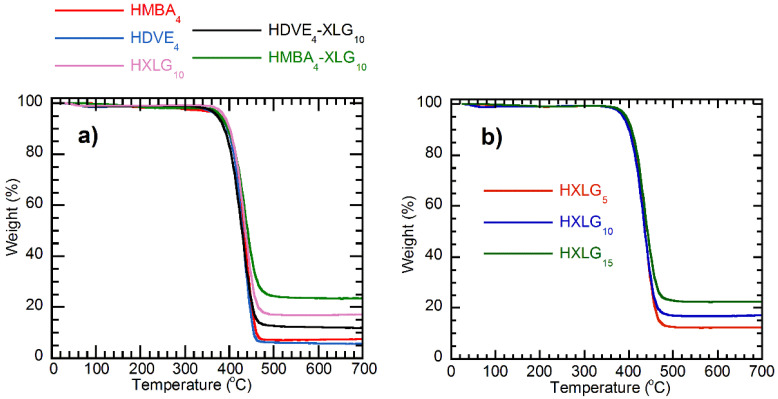
TGA plots for the hydrogels obtained: (**a**) the effect of the crosslinker type; (**b**) the effect of the XLG concentration.

The TGA investigation of the synthesized xerogels showed a unique decomposition step starting at about 350 °C for both NC and MBA/DVE-crosslinked hydrogels, as well as the presence of a variable amount of residue for all samples (Figure 7). The addition of the clay did not change the thermal stability of the PNVP matrix, as can be seen from the decomposition temperature being relatively close for all samples, irrespective of the presence of XLG (Figure 7a) or its amount (Figure 7b) within the xerogel. The fully organic xerogels did not completely decompose at 700 °C, leaving similar residue amounts of about 6% in the case of HDVE_4_ and 7.5% for HMBA_4_. The clay addition to the hydrogel led to an increase in the residue amount, regardless of XLG being the only crosslinker or a co-crosslinker together with MBA or DVE (Figure 7a), which additionally demonstrated its presence within the hydrogel. Additionally, by adding increasing amounts of XLG to the hydrogel, the xerogel residue increased as well (Figure 7b).

Besides TEM, another piece of direct evidence of the presence of the clay within the NC hydrogels was provided by EDX microanalysis. The EDX investigation of the swelled and lyophilized hydrogels showed the presence of silicon, aluminum and magnesium atoms from XLG within the NC hydrogels, along with the nitrogen, carbon and oxygen atoms belonging to the PNVP matrix (Figure S2). Additionally, the uniform dispersion of the clay within the hydrogel was demonstrated (Figure S2b,c).

### 3.2. Equilibrium Swelling Degree

**Figure 8:**
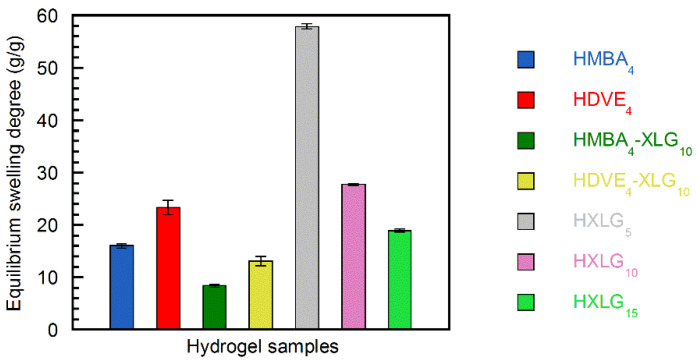
Equilibrium swelling degrees of the hydrogel samples synthesized.

The equilibrium swelling degree (ESD) measurements (Figure 8) were in agreement with the results and explanations from the gel fractions determination. Thus, the lower ESD of HMBA_4_ (16.0 ± 0.4 g/g) in comparison with HDVE_4_ (23.3 ± 1.4 g/g) proved the higher crosslinking degree of HMBA_4_, as explained above based on the higher reactivity of MBA in the copolymerization reaction with NVP. The addition of 10 wt% XLG based on NVP to the divinyl monomer–containing hydrogel compositions (HMBA_4_-XLG_10_, HDVE_4_-XLG_10_) increased both the gel fraction (Figure 1) and the crosslinking density of the hydrogels owing to the crosslinking effect of XLG. This resulted in an ESD decrease in both cases (8.4 ± 0.3 g/g HMBA_4_-XLG_10_; 13.1 ± 0.9 g/g HDVE_4_-XLG_10_, Figure 8) in comparison with the ESD of both HXLG_10_ (27.7 ± 0.2 g/g) and the corresponding divinyl monomer-crosslinked hydrogel, as expected. Similar to the HMBA_4_ vs. HDVE_4_ case, the ESD of HMBA_4_-XLG_10_ was lower than that of HDVE_4_-XLG_10_, suggesting that the presence of MBA led to a higher degree of crosslinking in this case as well.

The NC hydrogels crosslinked with various XLG amounts displayed ESDs inversely proportional with the clay percentage (57.9 ± 0.5 g/g HXLG_5_ > 27.7 ± 0.2 g/g HXLG_10_ > 18.9 ± 0.3 g/g HXLG_15_), indicating an increase in the crosslinking density from HXLG_5_ to HXLG_15_ (Figure 8) [73]. The same crosslinking density increase was supported by the gel fraction modification in the order GF_HXLG5_ < GF_HXLG10_ < GF_HXLG15_ (Figure 1). A lower gel fraction is indicative of the formation of a hydrogel network with a larger proportion of defects, such as intramolecular loops and free chain ends, leading to a smaller concentration of elastically effective chains and, therefore, to a lower network density and a higher ESD [74,75].

### 3.3. Viscoelastic Properties

**Figure 9:**
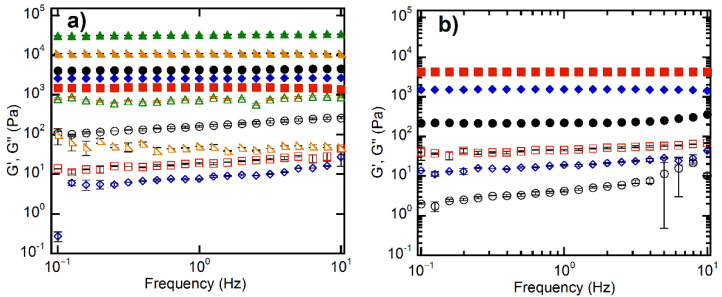
Frequency sweep rheological measurements of the hydrogel samples synthesized. (**a**) The effect of crosslinker type. HMBA_4_ (●,○); HDVE_4_ (◆,◇); HXLG_10_ (■,□); HMBA_4_-XLG_10_ (▲,△); HDVE_4_-XLG_10_ (◣,◺). (**b**) The effect of XLG concentration. HXLG_5_ (●,○); HXLG_10_ (◆,◇); HXLG_15_ (■,□). G' = full symbols; G" = empty symbols.

**Figure 10:**
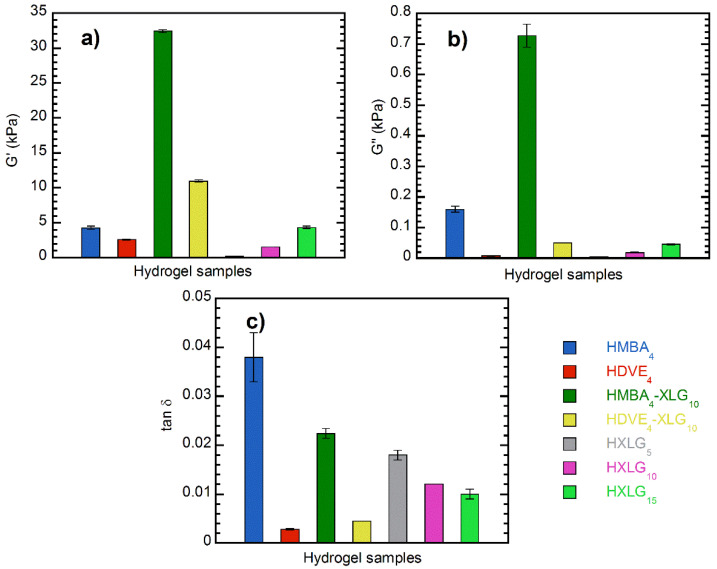
Frequency sweep rheological measurements of the hydrogel samples synthesized: (**a**) storage modulus; (**b**) loss modulus; (**c**) loss factor. Values recorded at 1 Hz frequency.

Rheological measurements of the equilibrium-swelled hydrogels were carried out at 25 °C in order to investigate their viscoelastic behavior. Frequency sweep experiments showed that the storage modulus G’ was higher than the loss modulus G” over the entire frequency range investigated for all hydrogels (Figure 9), which, together with the loss factor being smaller than the unity (Figure 10c), proved the crosslinked character of the samples.

According to the phantom model adapted by Peppas and coworkers [76] to real swollen hydrogels, G’ is, besides other network parameters, directly proportional to the polymer fraction in the swelled hydrogel to the 1/3 power and inversely proportional to the number average molecular weight between crosslinks (M_c_). A lower polymer fraction means a higher swelling degree, while a smaller M_c_ is equivalent to a higher degree of crosslinking/network density. Therefore, G’ should be smaller for higher swelling degrees as well as for lower crosslinking degrees/network densities and larger for reduced swelling degrees and higher crosslinking degrees/network densities. Additionally, the crosslinking degree and the ESD of hydrogels are connected each other, as we mentioned before. The G’ value of HMBA_4_ (4.27 ± 0.25 kPa at 1 Hz) was larger than that of HDVE_4_ (2.60 ± 0.06 kPa) (Figure 9a and Figure 10a), which is in agreement with both its higher crosslinking degree and lower ESD (Figure 8) [76,77]. However, HMBA_4_ displayed a greater viscous character in comparison with HDVE_4_, as demonstrated by its much larger loss factor (0.038 ± 0.005 vs. 0.0028 ± 0.0001, Figure 10c), which was probably a consequence of its less homogeneous character. The NC hydrogels synthesized in the presence of only XLG (HXLG_5-10-15_) displayed increasing G’ as the clay concentration increased (Figure 9b and Figure 10a,c), in accordance with the evolution of the hydrogel network density and swelling degree (Figure 8) [73,76,77] and the decreasing tan δ values. The higher loss factor at lower clay concentrations may be explained by the less homogeneous network, as indicated by the lower gel fraction (Figure 1). As explained above, a lower gel fraction is indicative of the formation of a hydrogel network with a larger proportion of defects, such as intramolecular loops and free chain ends [74,75]. The storage modulus of HXLG_15_ (4.32 ± 0.17 Pa) was practically equal to that of HMBA_4_ (4.27 ± 0.25 Pa), but its loss factor was appreciably lower (0.010 ± 0.001 vs. 0.038 ± 0.005), indicative of a more elastic character (Figure 10c), probably due to the more homogeneous network. However, in comparison with HDVE_4_ (tan δ = 0.0028 ± 0.0001), the loss factor of HXLG_15_ was about 4 times higher, suggesting a more viscous character of the clay-crosslinked hydrogel, although its G’ value was larger (4.32 ± 0.17 kPa vs. 2.60 ± 0.06 kPa).

The addition of 10 wt% XLG to NVP, along with the divinyl monomer, determined an increase in the number of crosslinking points within the hydrogel, which led to much larger G’ values (Figure 9a and Figure 10a). The enhancement was much more pronounced for the HMBA_4_-XLG_10_ hydrogel, resulting in a G’ value (32.40 ± 0.21 kPa) about 8 times higher than that of HMBA_4_ (4.27 ± 0.25 Pa), in comparison with a 4-times increase for HDVE_4_-XLG_10_ (10.96 ± 0.18 kPa), as compared to HDVE_4_ (2.60 ± 0.06 kPa). However, despite its lower G’ value, HDVE_4_-XLG_10_ displayed a more elastic character than HMBA_4_-XLG_10_, as demonstrated by the smaller loss factor (0.0045 ± 0.0000 vs. 0.022 ± 0.001, Figure 10c), which is in agreement with its more homogeneous network. It is interesting to point out that XLG addition led to a decrease in tan δ and, therefore, to an increase in the elastic character for HMBA_4_-XLG_10_, while in the case of HDVE_4_-XLG_10_, a slight increase in tan δ and, therefore, a decrease in the elastic character as compared with HDVE_4_ occurred (Figure 10c). This seems to indicate that the addition of XLG improved the homogeneity of the HMBA_4_-XLG_10_ network as compared to that of HMBA_4_, while in the case of HDVE_4_-XLG_10_, the network homogeneity was slightly disturbed by the clay in comparison with HDVE_4_.

### 3.4. Compressive Mechanical Properties

**Figure 11:**
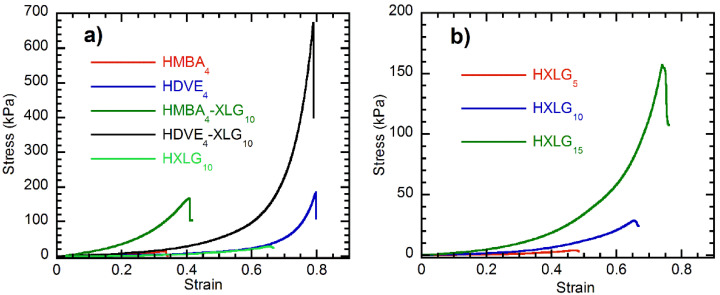
Typical stress–strain curves for the hydrogels synthesized: (**a**) the effect of crosslinker type; (**b**) the effect of XLG concentration.

The mechanical properties of the synthesized hydrogels were investigated by means of uniaxial compressive tests. Equilibrium-swelled cylindrical hydrogel samples were tested at room temperature, and both the elastic (E) and shear (G) moduli, the ultimate compressive stress (τ_max_) and the corresponding ultimate compressive strain (1-λ)_max_, as well as the compressive toughness (T_c_), were determined as the mean values of five-measurements sets. The graphs in Figure 11 display the curves with the closest parameters to those average values. In all cases, the ultimate compressive stress was equal to the stress value at which the hydrogel fractured.

**Figure 12:**
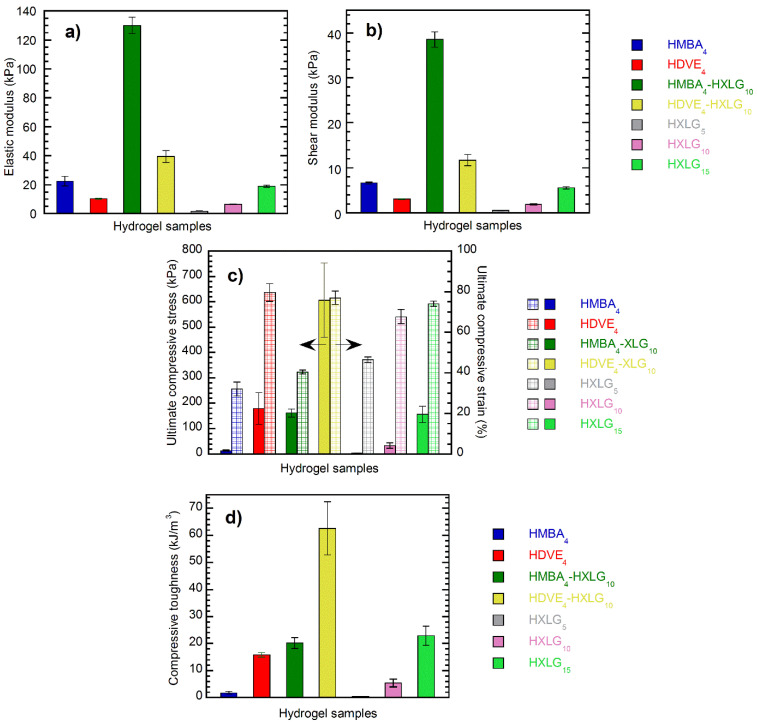
Compressive mechanical properties of the hydrogels synthesized: (**a**) elastic modulus; (**b**) shear modulus; (**c**) ultimate compressive stress and strain; (**d**) compressive toughness.

The values of both the elastic (E) and compressive shear (G) moduli of the hydrogels displayed the same order, i.e., HMBA_4_-XLG_10_ > HDVE_4_-XLG_10_ > HMBA_4_ > HXLG_15_ > HDVE_4_ > HXLG_10_ > HXLG_5_ (Figure 12a,b), as that in the case of the storage modulus G’ obtained by rheological measurements (Figure 10a). The elastic modulus of the hydrogels synthesized ranged between 1.66 ± 0.19 kPa for HXLG_5_ and 129.99 ± 5.71 kPa for HMBA_4_-XLG_10_, while their G values obtained by compressive tests, lying within the 0.49 ± 0.06–38.55 ± 1.69 kPa range, were in reasonably good agreement with the corresponding G’ values (Table S1), confirming the accuracy of the experiments. Therefore, the E and G moduli obtained by compressive mechanical tests validated the conclusions of the rheological measurements regarding the crosslinking degree of the synthesized hydrogels. One should also keep in mind that, similar to the G’ case, the hydrogels crosslinked by both the clay and divinyl monomer, i.e., HMBA_4_-XLG_10_ and HDVE_4_-XLG_10_, displayed the largest E and G, but the HMBA_4_-XLG_10_ values were about 3 times higher than those of HDVE_4_-XLG_10_ (Table S2, Figure 12a,b).

The E/G ratio was 3.37–3.39 (Table S2), which was higher than the expected value (E/G = 3) for rubbery materials such as the fully swelled hydrogels [78,79]. However, an E/G ratio higher than 3 is typically obtained in the case of uniaxial compressive measurements carried out on hydrogels [60,79] and depends on the strain interval employed to determine G, as predicted by Equation (6) [79]:

G = E/(1 + 2λ^−3^) (6)

According to Equation (6), an E/G ratio of 3 is obtained at very low deformations, when λ→1 [79]. Therefore, the larger E/G values obtained in our case were due to the 0.97–0.92 deformation interval we used in our calculations in order to ensure a linear stress–strain dependency.

The compression measurements also showed that, as far as the ultimate compressive strength was concerned (Figure 12c), the order (HDVE_4_-XLG_10_ > HDVE_4_ ≈ HMBA_4_-XLG_10_ ≈ HXLG_15_ > HXLG_10_ > HMBA_4_ > HXLG_5_) was quite different than that noticed in the case of E and G (Figure 12a,b and Figure S3). One can see that the highest τ_max_ was displayed by HDVE_4_-XLG_10_ (606.74 ± 141.71 kPa), which, unlike the corresponding E and G values, was about 4 times larger than that for HMBA_4_-XLG_10_ (162.04 ± 16.06 kPa). Additionally, HDVE_4_ (τ_max_ = 179.58 ± 62.2 kPa) displayed an ultimate compressive strength similar to that of HMBA_4_-XLG_10_ and HXLG_15_ (τ_max_ = 156.03 ± 32.65 kPa) and much larger (about 11 times) than that of HMBA_4_ (τ_max_ = 14.07 ± 2.75 kPa), which was totally different than that for the corresponding elastic moduli (E_HDVE4_ = 10.26 ± 1.20 kPa < E_HXLG15_ = 18.79 ± 0.87 kPa < E_HMBA4_ = 22.35 ± 0.81 kPa < E_HMBA4-XLG10_ = 129.99 ± 5.71 kPa). In the case of hydrogels crosslinked only by XLG (HXLG_5-10-15_), the clay content increase led to progressively higher τ_max_ values (Figure 12c), i.e., 3.61 ± 0.80 kPa for 5 wt% XLG, 33.79 ± 10.49 kPa for 10 wt% XLG and 156.03 ± 32.65 kPa for 15 wt% XLG.

Besides the E and G moduli, the ultimate compressive strength depends on the corresponding ultimate compressive strain ((1−λ)_max_) as well, which may be considered a measure of the flexibility of the sample. The results showed that the (1−λ)_max_ values (Figure 12c) of the hydrogels strictly followed the opposite order of the rheometrically determined loss factor (Figure 10c), i.e., HDVE_4_ > HDVE_4_-XLG_10_ > HXLG_15_ > HXLG_10_ > HXLG_5_ > HMBA_4_-XLG_10_ > HMBA_4_ for (1−λ)_max_ vs. HDVE_4_ < HDVE_4_-XLG_10_ < HXLG_15_ < HXLG_10_ < HXLG_5_ < HMBA_4_-XLG_10_ < HMBA_4_ in the case of tan δ (Figure S4). Taking into account that a lower value of the loss factor is indicative of a higher elastic/lower viscous character of a hydrogel, it results in the larger (1−λ)_max_ values being displayed by the more elastic samples with a more uniform network, while the more viscous–less homogeneous-network ones showed smaller ultimate compressive strains.

We also calculated the compressive toughness (T_c_) of the hydrogels (Figure 12d), which is a measure of the energy that a material can absorb before failure—that is, a measure of its resistance to destruction [80]. The largest T_c_ value was displayed by HDVE_4_-XLG_10_ (62.55 ± 9.87 kJ/m^3^) because of its homogeneous network and high crosslinking density, while HMBA_4_-XLG_10_, with a less homogeneous network but a larger crosslinking density, showed a T_c_ value (20.17 ± 2.02 kJ/m^3^) about 3 times lower. Additionally, the compressive toughness of HXLG_15_ (22.93 ± 3.56 kJ/m^3^) was slightly higher than that of HMBA_4_-XLG_10_ (20.17 ± 2.02 kJ/m^3^), most likely because of the more homogeneous network, although its crosslinking density was much lower, as proved by its much larger ESD (Figure 8) and lower G and G’ (Table S1). Similarly, HDVE_4_, with a more homogeneous but less crosslinked network, had a T_c_ (15.79 ± 3.65 kJ/m^3^) about 9 times higher than that of HMBA_4_ (1.74 ± 0.48 kJ/m^3^). The addition of 10 wt% XLG to the divinyl monomer-crosslinked hydrogels led to an 11.6-times increase in T_c_ in the case of HMBA_4_-XLG_10_ in comparison to a roughly 4-times increase for HDVE_4_-XLG_10_. This difference may be explained by the fact that both the network homogeneity and density increased in the case of HMBA_4_-XLG_10_, while for HDVE_4_-XLG_10_, the crosslinking density increased while the network homogeneity slightly decreased, as discussed before. In the case of the clay-only-crosslinked hydrogels, T_c_ progressively increased from HXLG_5_ to HXLG_15_ (0.46 ± 0.07 kJ/m^3^ < 5.44 ± 1.41 kJ/m^3^ < 22.93 ± 3.56 kJ/m^3^, Figure 12c), in agreement with the increase in both the crosslinking density and the network homogeneity in the same direction.

## 4. Conclusions

DVE proved to be a much better crosslinking monomer for the synthesis of PNVP hydrogels than MBA, as it allowed for the obtention of a more homogeneous, although less dense, network. This was a consequence of DVE, unlike MBA, having a similar reactivity to that of NVP in the copolymerization reaction. Because of this, HDVE_4_ displayed a larger gel fraction, ESD, ultimate compressive stress and strain and compressive toughness and a higher elastic character in comparison with HMBA_4_, but it displayed a lower crosslinking density and lower G’/G and E moduli. A homogeneous network also resulted in the case of the XLG-only-crosslinked PNVP hydrogels, leading, in the case of HXLG_10_ and HXLG_15_, to viscoelastic and compressive mechanical properties similar to those of HDVE_4_ under the experimental conditions employed. However, different synthesis conditions, allowing for a larger XLG content of hydrogels, may lead to much better properties of the NC hydrogels, as suggested by the steady improvement of the HXLG_5-10-15_ properties with the increasing XLG content. The addition of XLG (10 wt%) as a second crosslinker together with the divinyl monomer (MBA/DVE) determined a strong increase in the gel fraction and the viscoelastic and compressive mechanical properties and a decrease in the ESD in comparison with the hydrogels crosslinked by only one of the crosslinkers tested (XLG/DVE/MBA). As compared to HDVE_4_-XLG_10_, HMBA_4_-XLG_10_ displayed much larger E and G’/G moduli, in agreement with its more crosslinked network, but a lower ultimate compressive stress and strain, compressive toughness and elastic character because of its less homogeneous network. The presence of the clay within the hydrogel improved the homogeneity of the HMBA_4_-XLG_10_ hydrogel network compared to that of HMBA_4_, while it seemed to slightly decrease the homogeneous character of the DVE-crosslinked PNVP network.

Laponite XLG displayed a uniform distribution within the NC hydrogels, as demonstrated by EDX analyses. The clay was mostly exfoliated, but platelet agglomerations, including more organized/non-exfoliated structures, were still present, as the TEM and XRD measurements showed. Two different clay–PNVP interactions, which are responsible for the crosslinking effect of Laponite, were evidenced by the splitting of the Si-O band of XLG at 967 cm^−1^ in the FTIR spectra of NC xerogels. Despite these interactions, the thermal stability of the dried hydrogels was not appreciably affected by the presence of clay, as revealed by TGA measurements.

## Figures and Tables

**Table 1:** The composition of the hydrogel preparation mixtures ^1^.

Sample	NVP(g)	MBA ^2^ (g)	DVE ^2^ (g)	AIBN ^3^ (g)	XLG ^4,5^ (g)	DW (g)
HMBA_4_	2.0	0.111	-	0.007	-	7.88
HDVE_4_	2.0	-	0.146	0.007	-	7.85
HMBA_4_-XLG_10_	2.0	0.111	-	0.007	0.200	7.68
HDVE_4_-XLG_10_	2.0	-	0.146	0.007	0.200	7.65
HXLG_5_	2.0	-	-	0.007	0.100	7.89
HXLG_10_	2.0	-	-	0.007	0.200	7.79
HXLG_15_	2.0	-	-	0.007	0.300	7.69

^1^ For 10 g of the polymerization mixture; ^2^ 4 mol% based on NVP; ^3^ 0.25 mol% based on NVP; ^4^ 5, 10 or 15 wt% based on NVP; ^5^ 0.24, 0.48 or 0.72 mol% based on NVP, calculated with M_XLG_ = 2286.9 g/mol [59].

## Data Availability

The data presented in this study are available on request from the corresponding author.

## Supplementary Materials

The following supporting information can be downloaded at: https://www.mdpi.com/article/10.3390/polym14194216/s1. Figure S1. The chemical structures of the vinyl monomers used. Figure S2. EDX analyses of the synthesized hydrogels. (a) HMBA_4_; (b) HXLG_10_; (c) HMBA_4_-XLG_10_. Figure S3. The descending order of the hydrogel samples from the point of view of (a) ultimate compressive stress and (b) elastic modulus. Figure S4. Comparison among the ultimate compressive strains and the loss factors of the synthesized hydrogels. Table S1. Comparison between the storage (G′) and compressive shear (G) moduli of the synthesized hydrogels. Table S2. The E (elastic modulus)/G (shear modulus) ratios for the synthesized hydrogels.
