# Supplementary material for: Poly(N-vinylpyrrolidone)–Laponite XLG Nanocomposite Hydrogels: Characterization, Properties and Comparison with Divinyl Monomer-Crosslinked Hydrogels
Source: Polymers (Basel). 2022 Oct 8;14(19):4216. doi: 10.3390/polym14194216 (PMC9571604; doi:10.3390/polym14194216)
Supplement: Supplementary file 1 [file polymers-14-04216-s001.zip › polymers-1938722-supplementary.pdf]

Supplementary Data

Poly(N-vinylpyrrolidone) - Laponite XLG nanocomposite hydrogels: characterization, properties and comparison with divinyl monomer-crosslinked hydrogels

Ionela Alice Podaru[1,2,#], Paul O. Stanescu[1,3,#], Raluca Ginghină[4], Stefania Stoleriu[5], Bogdan Trică[6], Raluca Somoghi[6,7], Mircea Teodorescu[1,*]

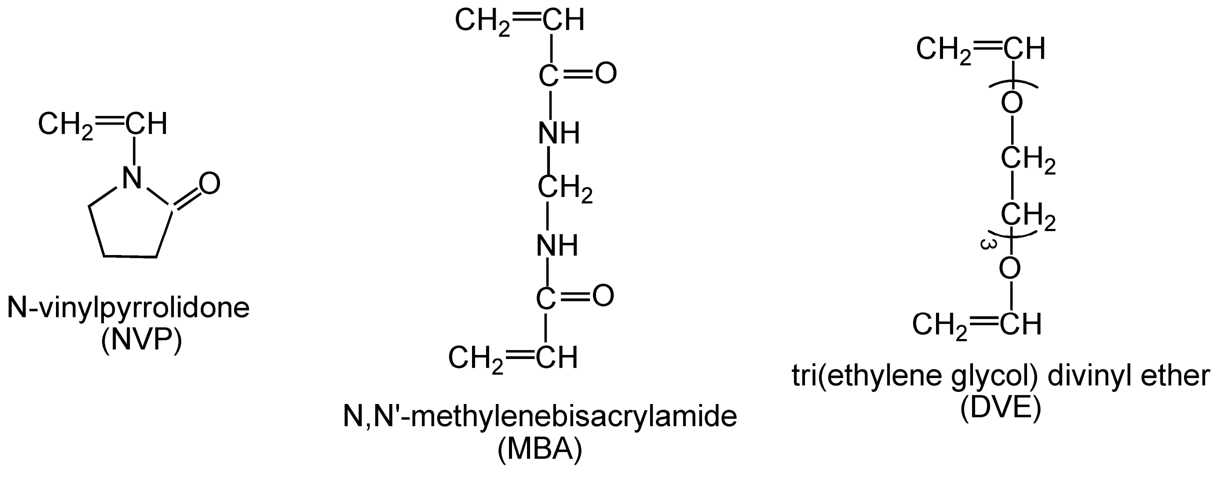

Figure S1. The chemical structures of the vinyl monomers used

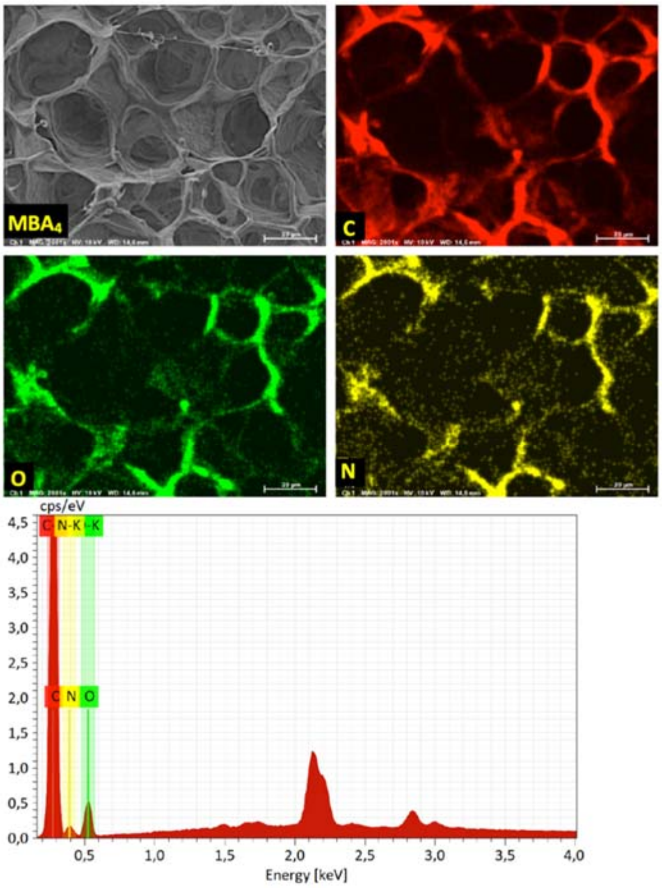

a)

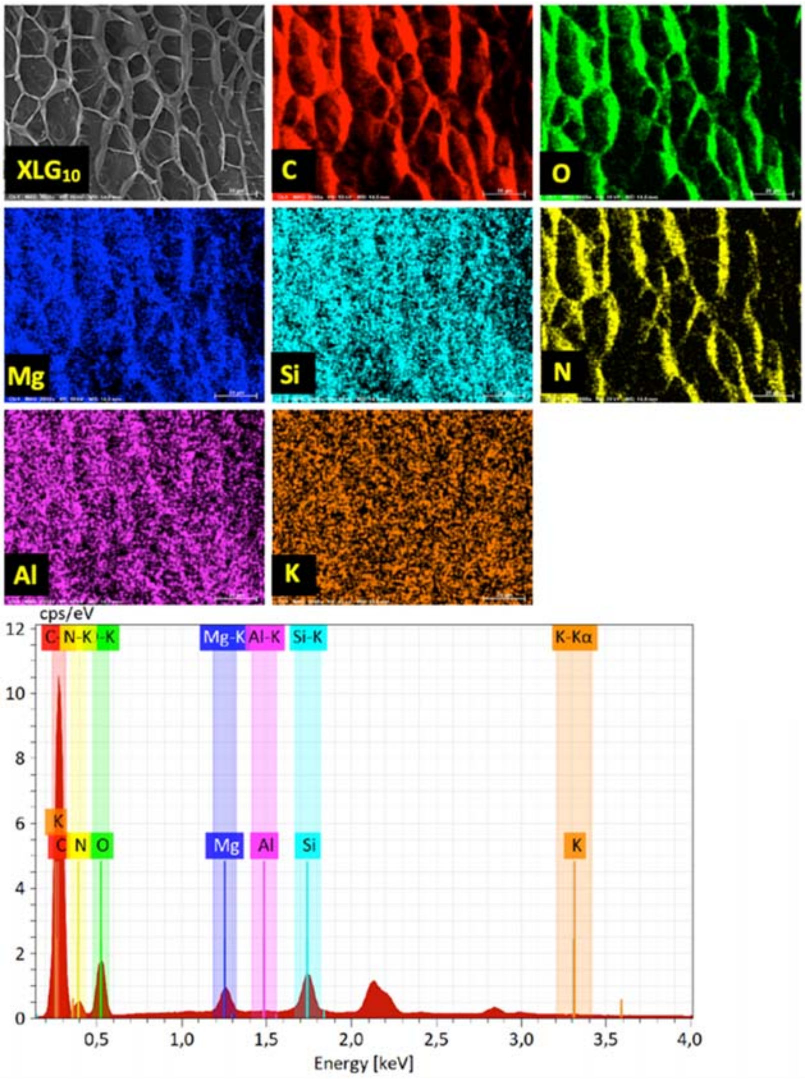
XLG10
C
O
Mg
Si
N
Al
K
cps/eV
C
N-K
-K
Mg-K
Al-K
Si-K
K-Kα
K
C
N
O
Mg
Al
Si
K
Energy [keV]

b)

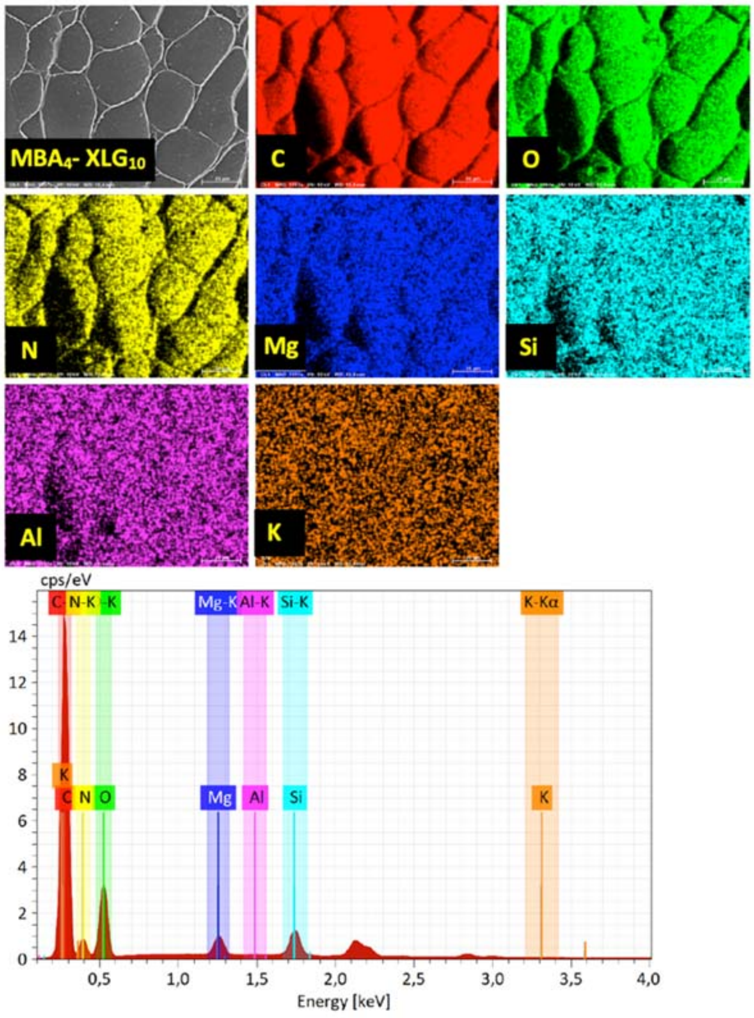

c)

Figure S2. EDX analyses of the of synthesized hydrogels. a) $HMBA_4$; b) $HXLG_{10}$; c) $HMBA_4$-XLG_{10}.

Table S1. Comparison between the storage (G') and compressive shear (G) moduli of the synthesized hydrogels

Modulus	Hydrogel sample						
	$HMBA_4$	$HDVE_4$	$HMBA_4$-XLG_{10}	$HDVE_4$-XLG_{10}	$HXLG_5$	$HXLG_{10}$	$HXLG_{15}$
G' (kPa) at 1 Hz	4.27±0.25	2.60±0.06	32.40±0.21	10.96±0.18	0.22±0.01	1.54±0.00	4.32±0.17
G (kPa)	6.63±0.24	3.04±0.35	38.55±1.69	11.72±1.22	0.49±0.06	1.89±0.05	5.57±0.26

Table S2. The E (elastic modulus)/G (shear modulus) ratios for the synthesized hydrogels

Modulus	Hydrogel sample						
	$HMBA_4$	$HDVE_4$	$HMBA_4$-XLG_{10}	$HDVE_4$-XLG_{10}	$HXLG_5$	$HXLG_{10}$	$HXLG_{15}$
E (kPa)	22.35±0.81	10.26±0.1.20	129.99±5.71	39.51±4.11	1.66±0.19	6.38±0.16	18.79±0.87
G (kPa)	6.63±0.24	3.04±0.35	38.55±1.69	11.72±1.22	0.49±0.06	1.89±0.05	5.57±0.26
E/G	3.37	3.37	3.37	3.37	3.39	3.37	3.37

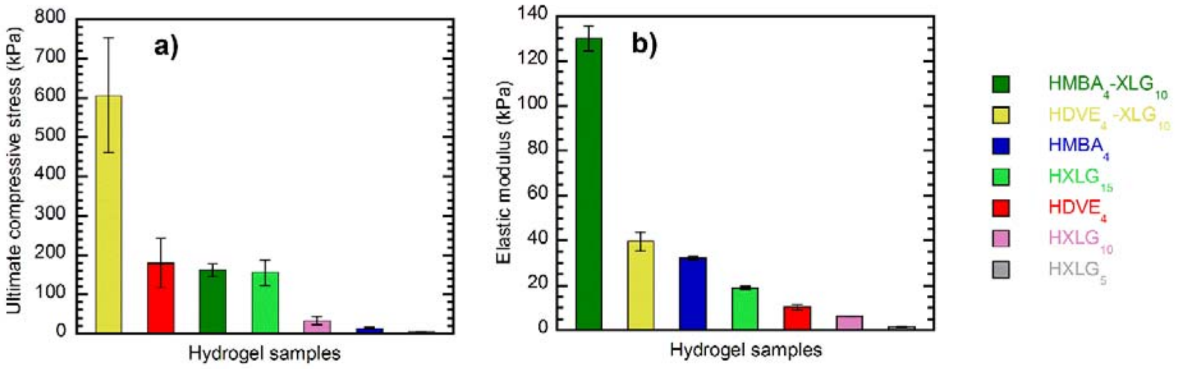

Figure S3. The descending order of the hydrogel samples from the point of view of a) ultimate compressive stress and b) elastic modulus.

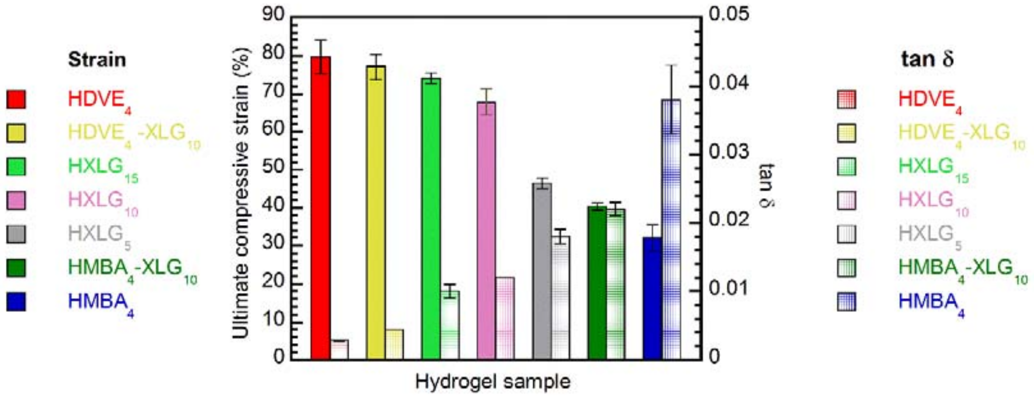

Figure S4. Comparison among the ultimate compressive strains and the loss factors of the synthesized hydrogels.
